# Supplementary material for: Antinociceptive Effect of Hinokinin and Kaurenoic Acid Isolated from Aristolochia odoratissima L
Source: Molecules. 2020 Mar 24;25(6):1454. doi: 10.3390/molecules25061454 (PMC7145305; doi:10.3390/molecules25061454)
Supplement: Supplementary file 1 [file molecules-25-01454-s001.pdf]

Article

Antinociceptive effect of hinokinin and kaurenoic acid isolated from *Aristolochia odoratissima* L.

Rosa Mariana Montiel-Ruiz [2], Marcos Córdova-de la Cruz [1], Manasés González-Cortázar [2], Alejandro Zamilpa [2], Abraham Gómez-Rivera [1], Ricardo López-Rodríguez [1], Carlos Ernesto Lobato-García [1] and Ever A. Blé-González [1*]

[1] Universidad Juárez Autónoma de Tabasco, Carretera Cunduacán-Jalpa Km. 0.5, 86690 Cunduacán, Tabasco, Mexico. E-Mails: everblesni@gmail.com (E.A.B.-G.); marcoscordovaqfb@gmail.com (M. C.-D.); abgori@gmail.com (A.G.-R.); carloslobatogarcia@gmail.com@gmail.com (C.E.L.-G.); Richard_lorr@hotmail.com (R.L.-R).

[2] Centro de Investigación Biomédica del Sur (CIBIS), Instituto Mexicano del Seguro Social (IMSS), Argentina No. 1, Col. Centro, 62790 Xochitepec, Morelos, Mexico. E-Mails: gmanases@hotmail.com (M.G.-C.); azamilpa_2000@yahoo.com.mx (A.Z.); montielrmariana@gmail.com (R.M.M.-R.).

* Correspondence: ble_49@hotmail.com
Phone: (+52) (993) 3581500, ext. 4711.

Received: date; Accepted: date; Published: date

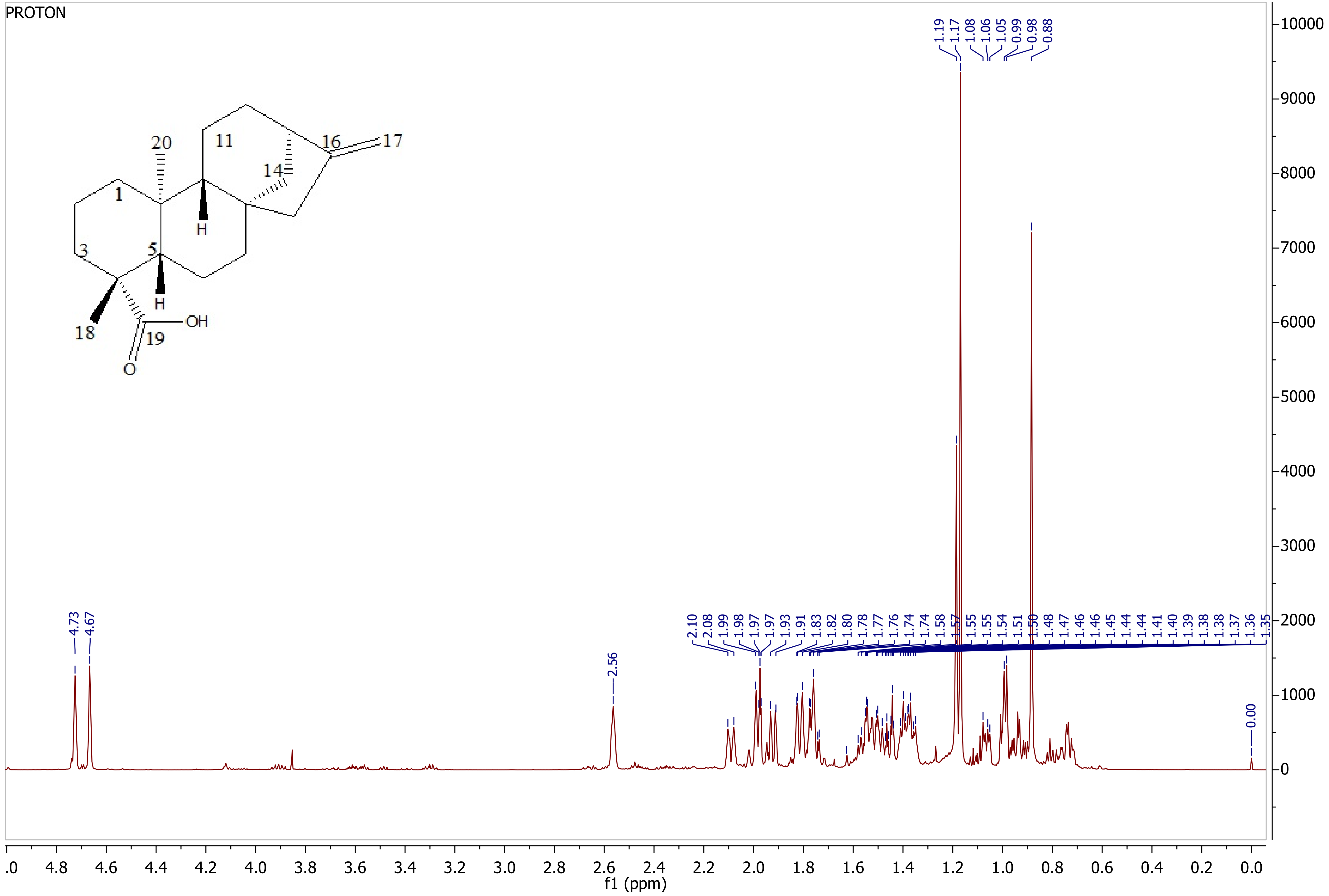

Figure S1. ^1H NMR ($CDCl_3$, 600 MHz) Compound **1**

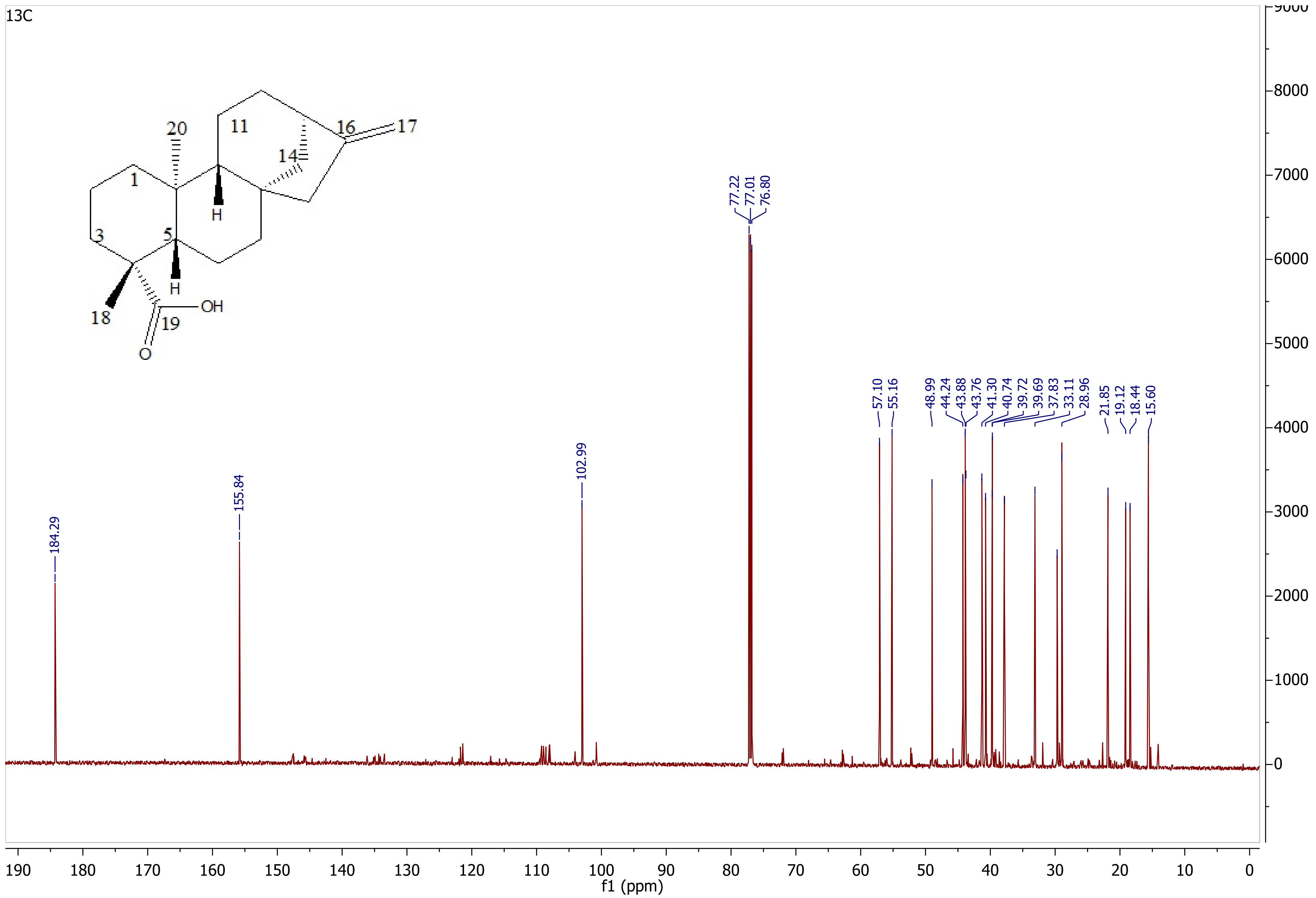

Figure S2. ^{13}C NMR ($CDCl_3$, 150 MHz) Compound **1**.

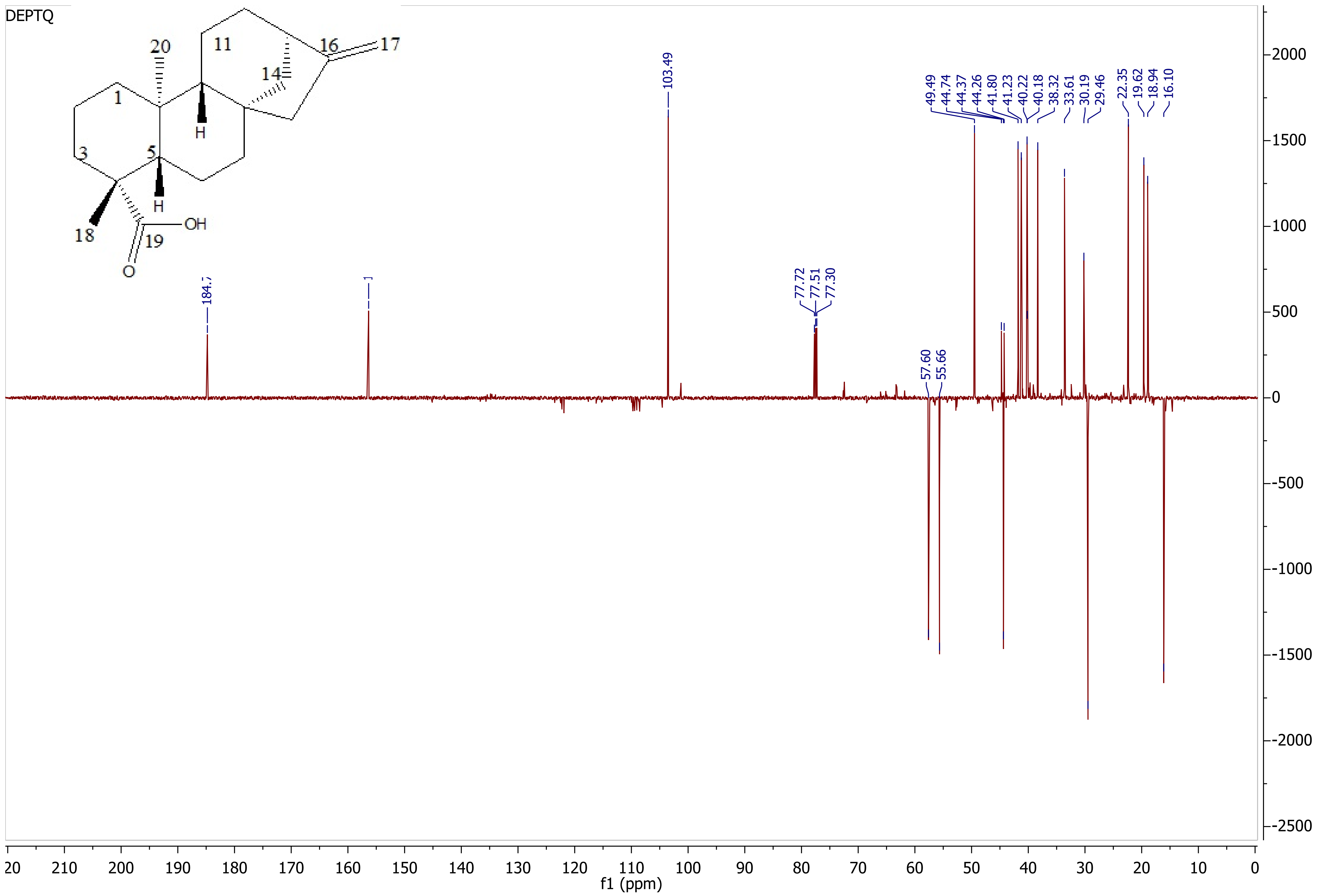

Figure S3. DEPTQ($CDCl_3$, 150 MHz) Compound **1**

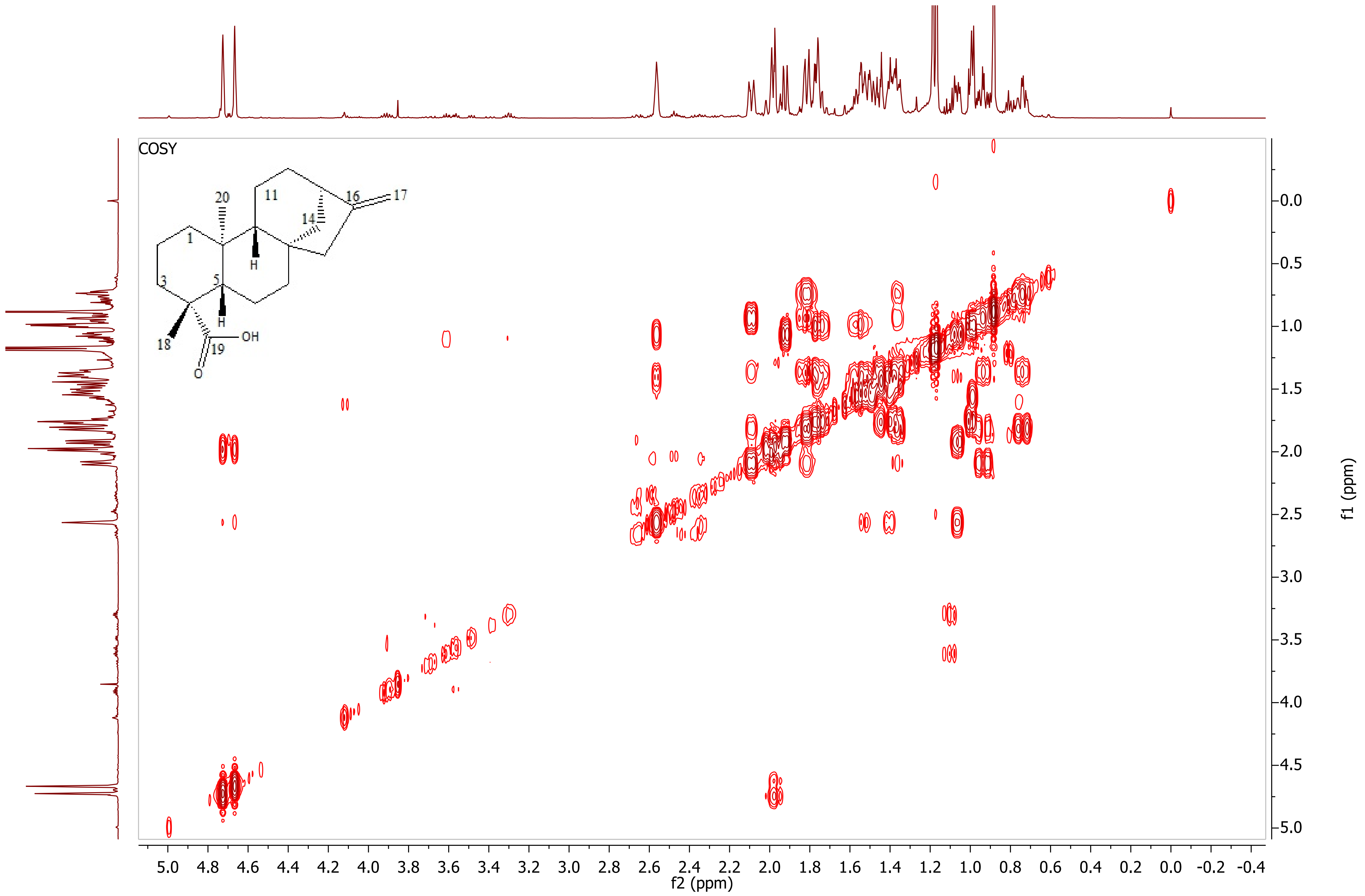

Figure S4. ^{1}H-^{1}H COSY NMR ($CDCl_3$, 600 MHz) Compound **1**

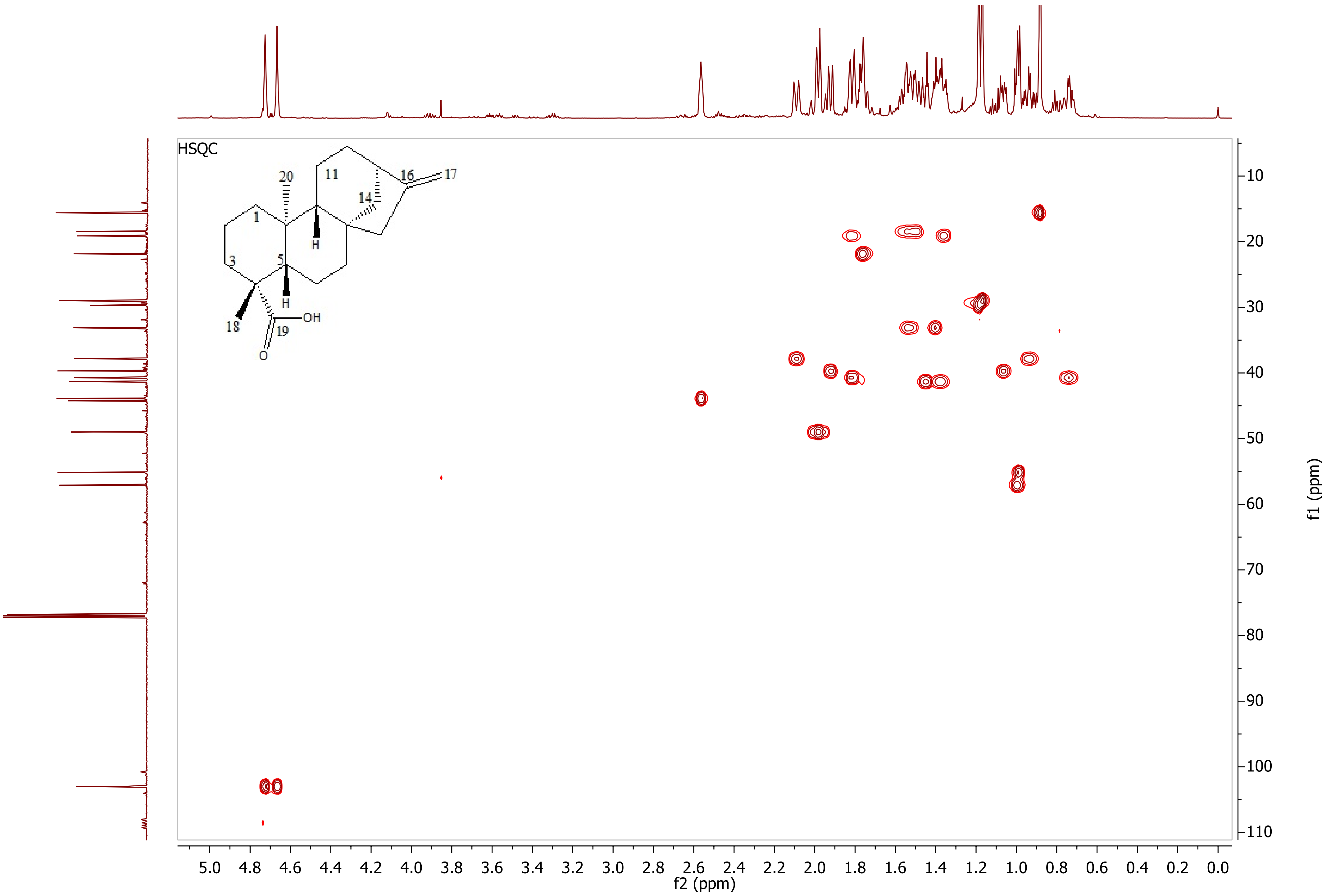

Figure S5. HSQC ($CDCl_3$, 600 MHz) Compound **1**.

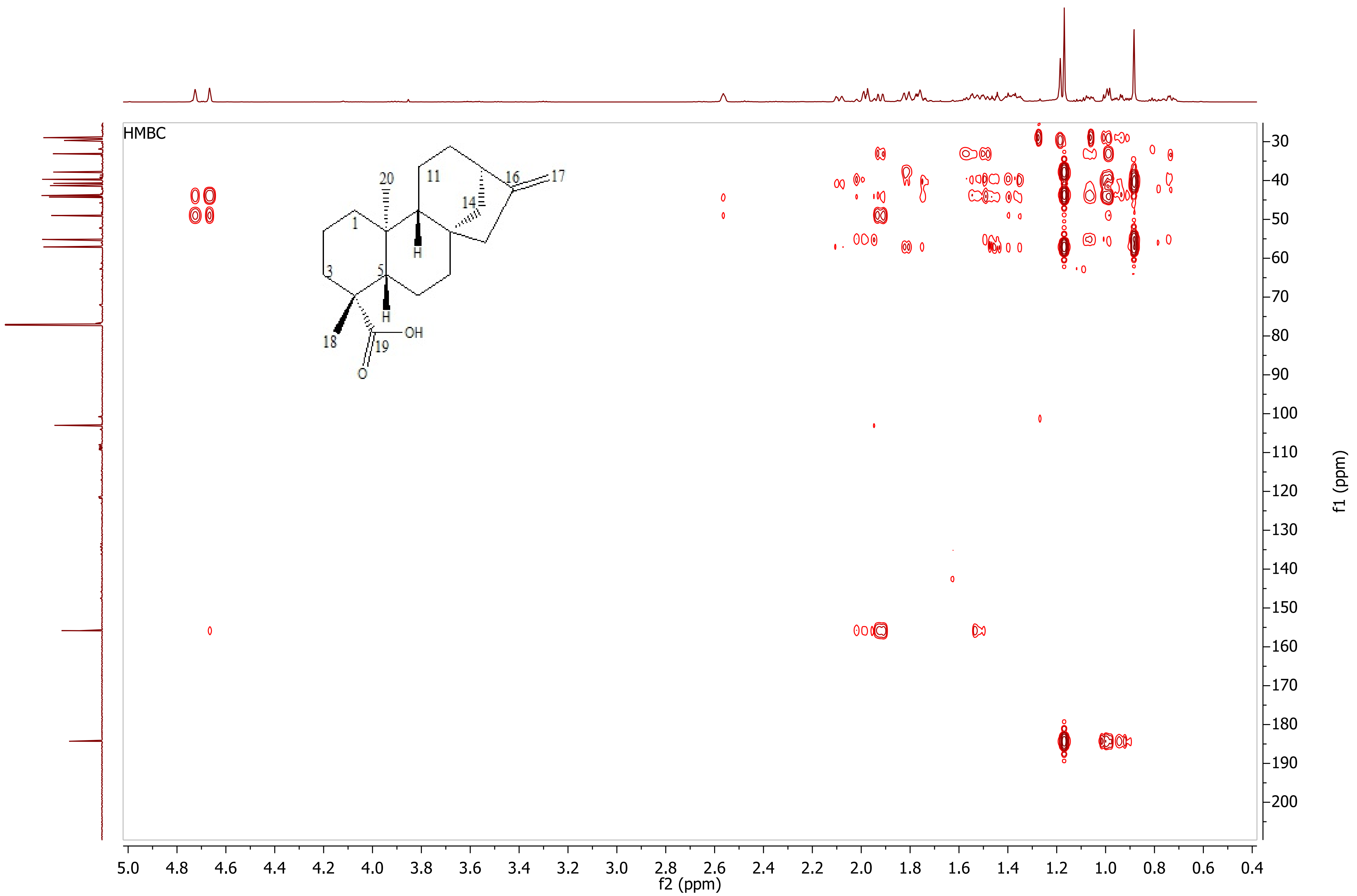

Figure S6. HMBC ($CDCl_3$, 600 MHz) Compound **1**

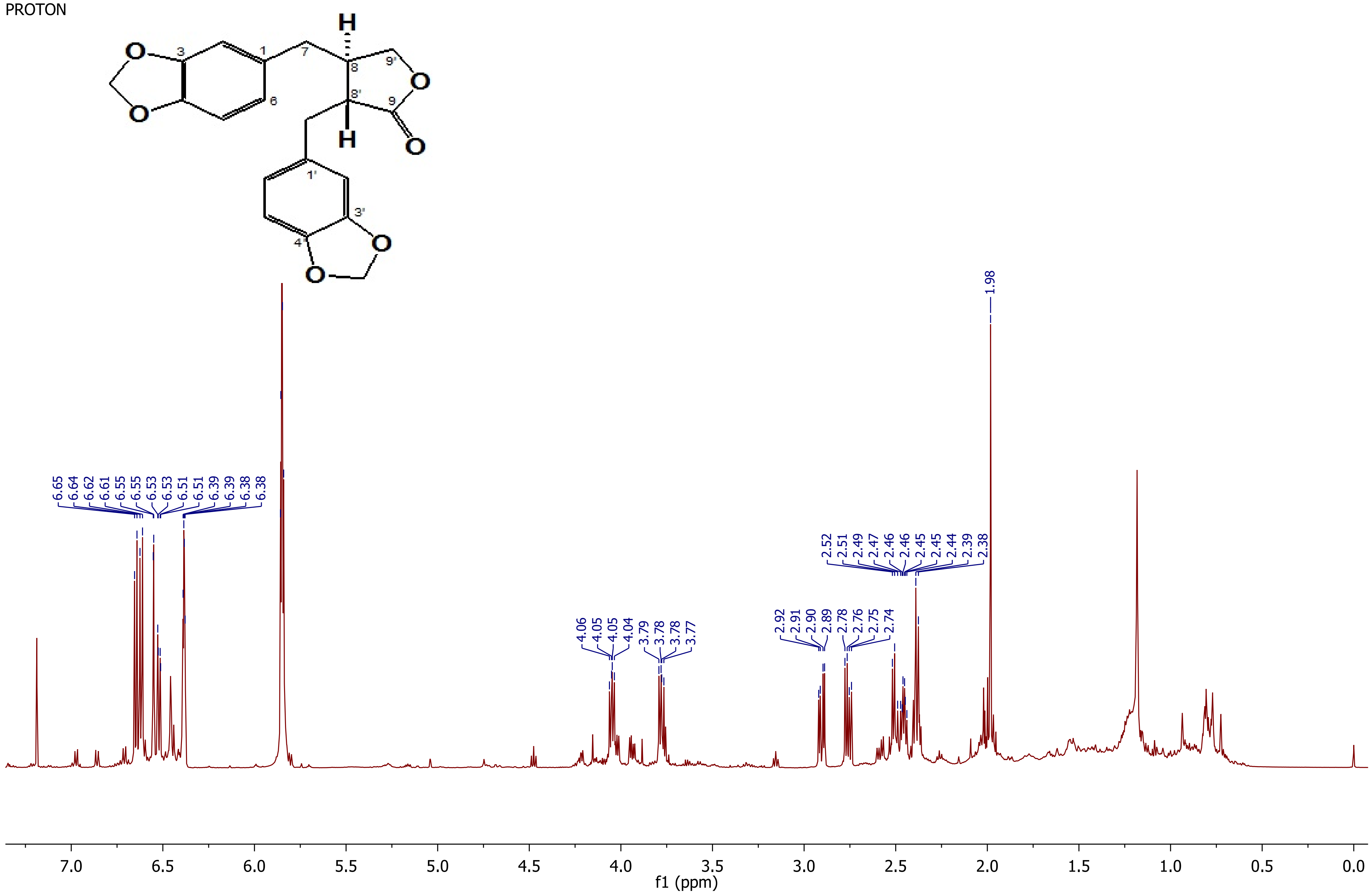

Figure S7. ^{1}H NMR ($CDCl_3$, 600 MHz) Compound **2.**

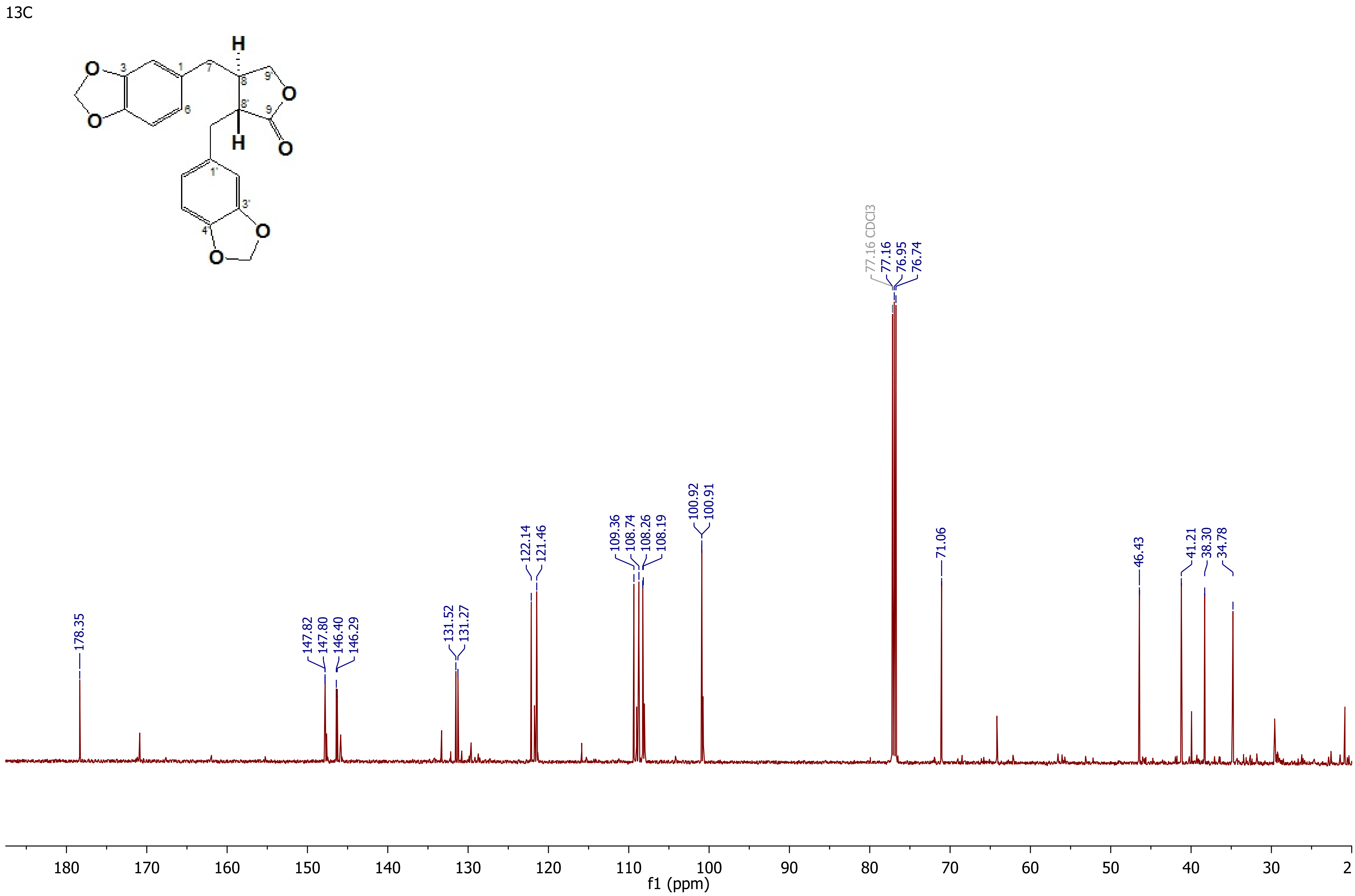

Figure S8. ^{13}C NMR ($CDCl_3$, 150 MHz) Compound **2**.

DEPTQ

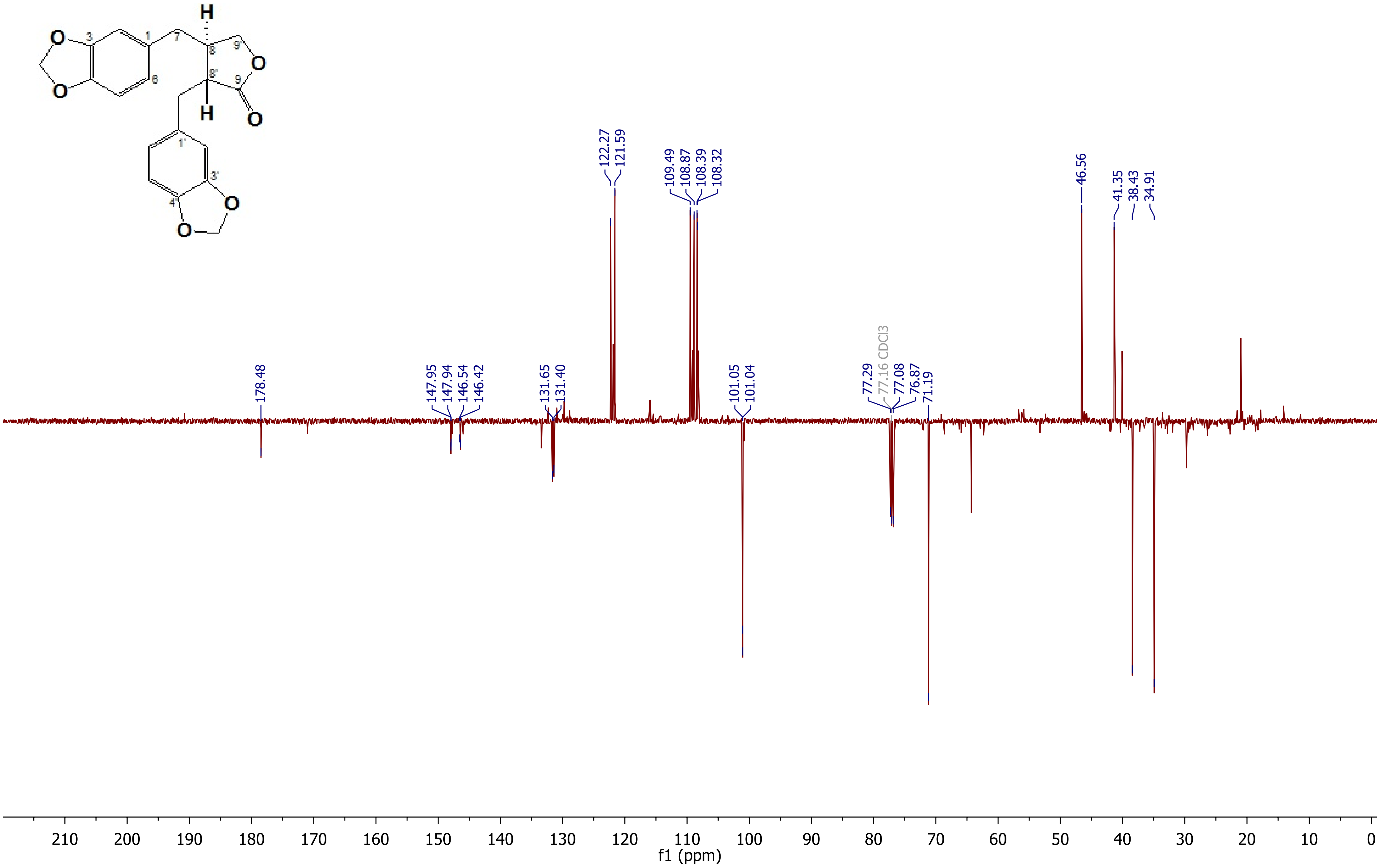

Figure S9. DEPTQ($CDCl_3$, 150 MHz) Compound **2**.

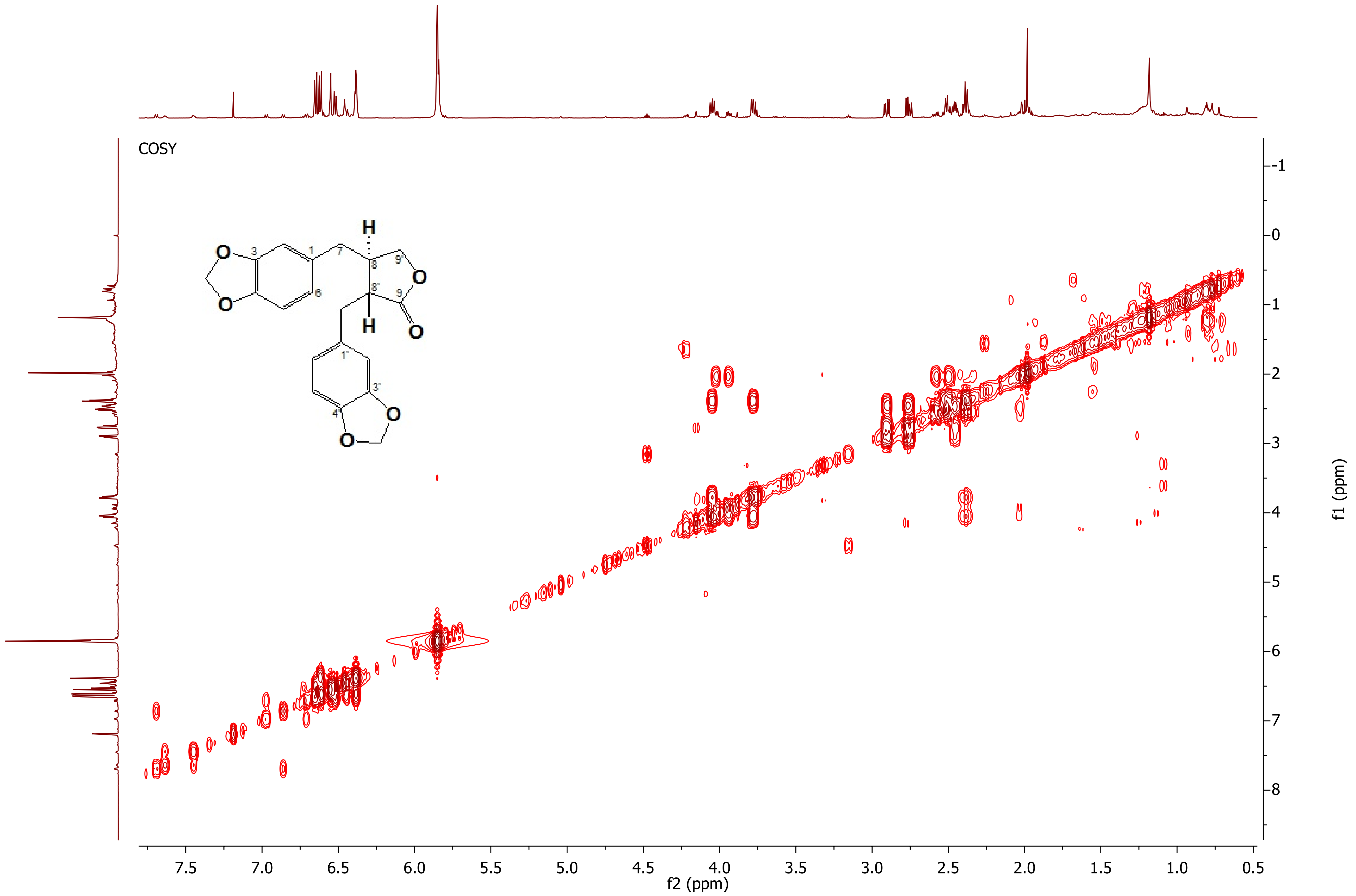

Figure S10. ^{1}H-^{1}H COSY NMR ($CDCl_3$, 600 MHz) Compound **2**.

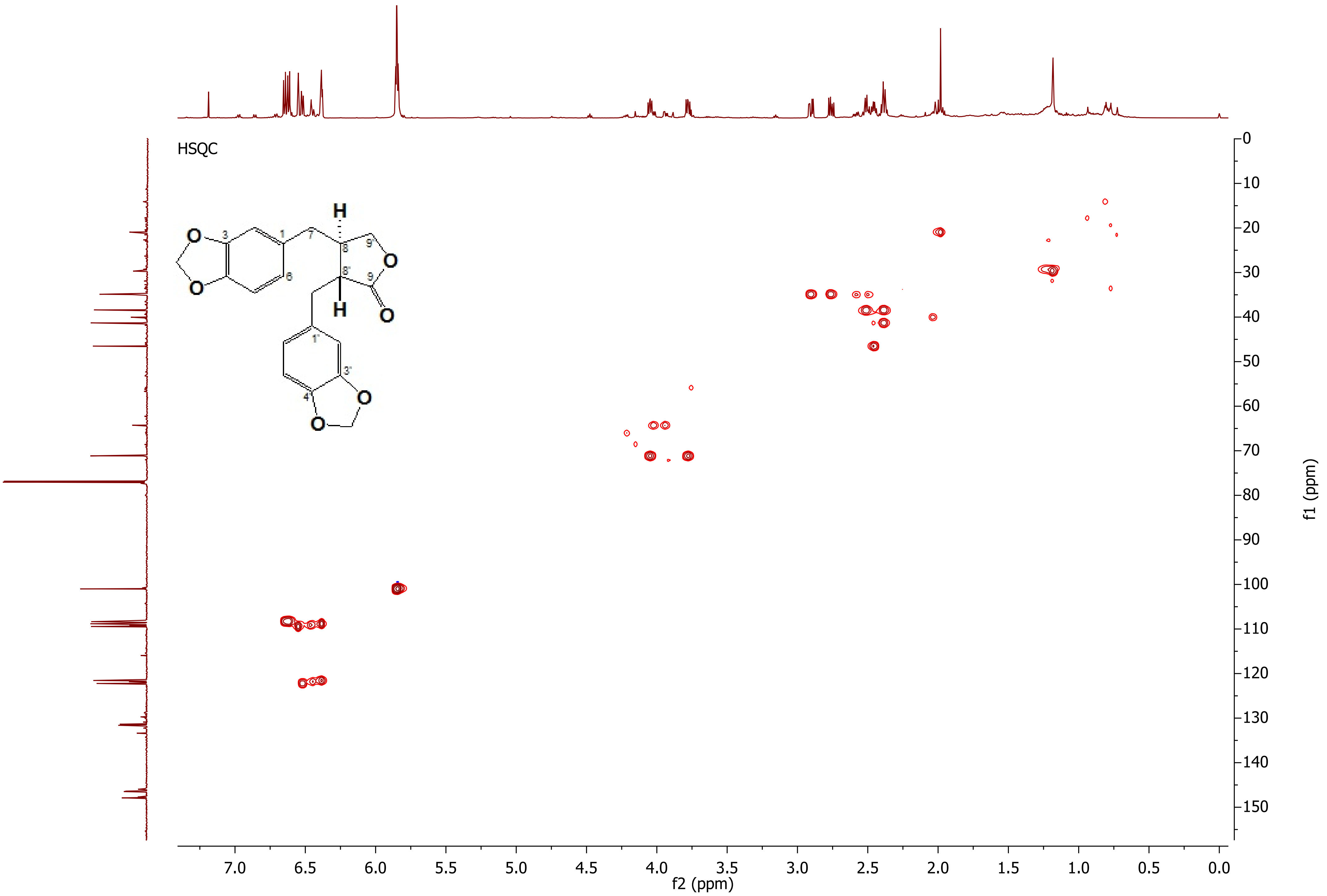

Figure S11. HSQC ($CDCl_3$, 600 MHz) Compound **2**.

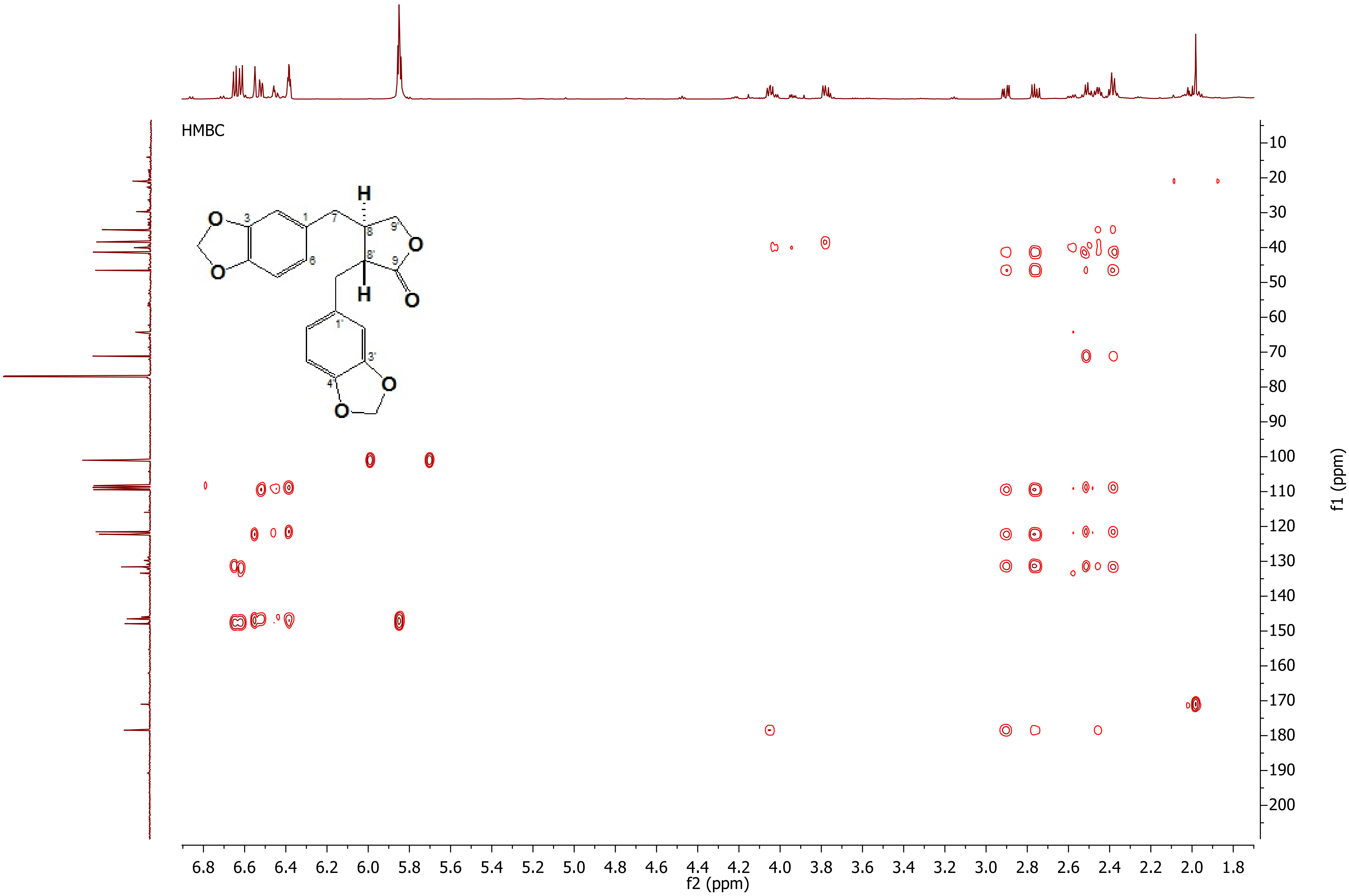

Figure S12. HMBC ($CDCl_3$, 600 MHz) Compound **2.**

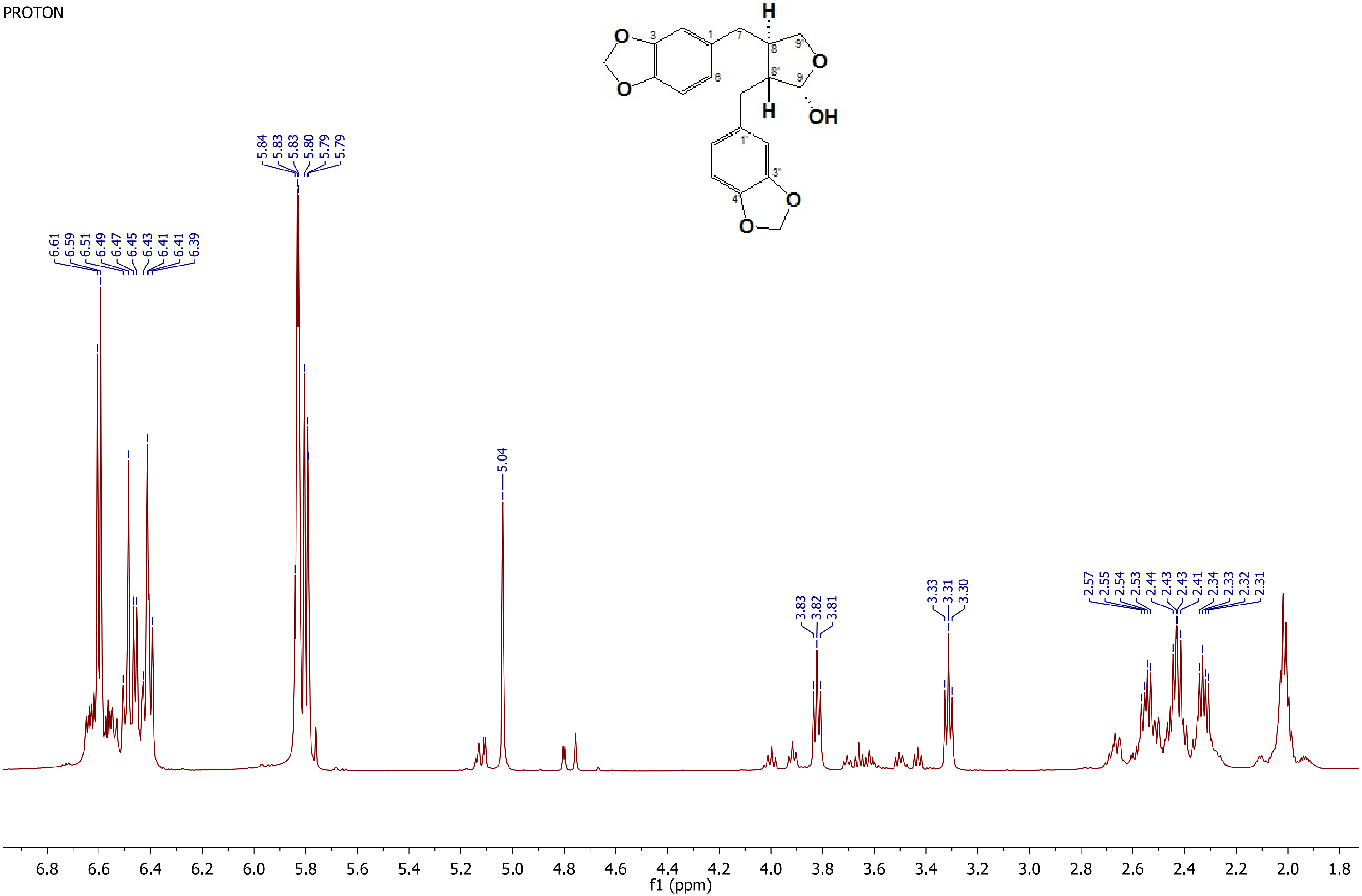

Figure S13. ^{1}H NMR ($CDCl_3$, 600 MHz) Compound **3**.

13C

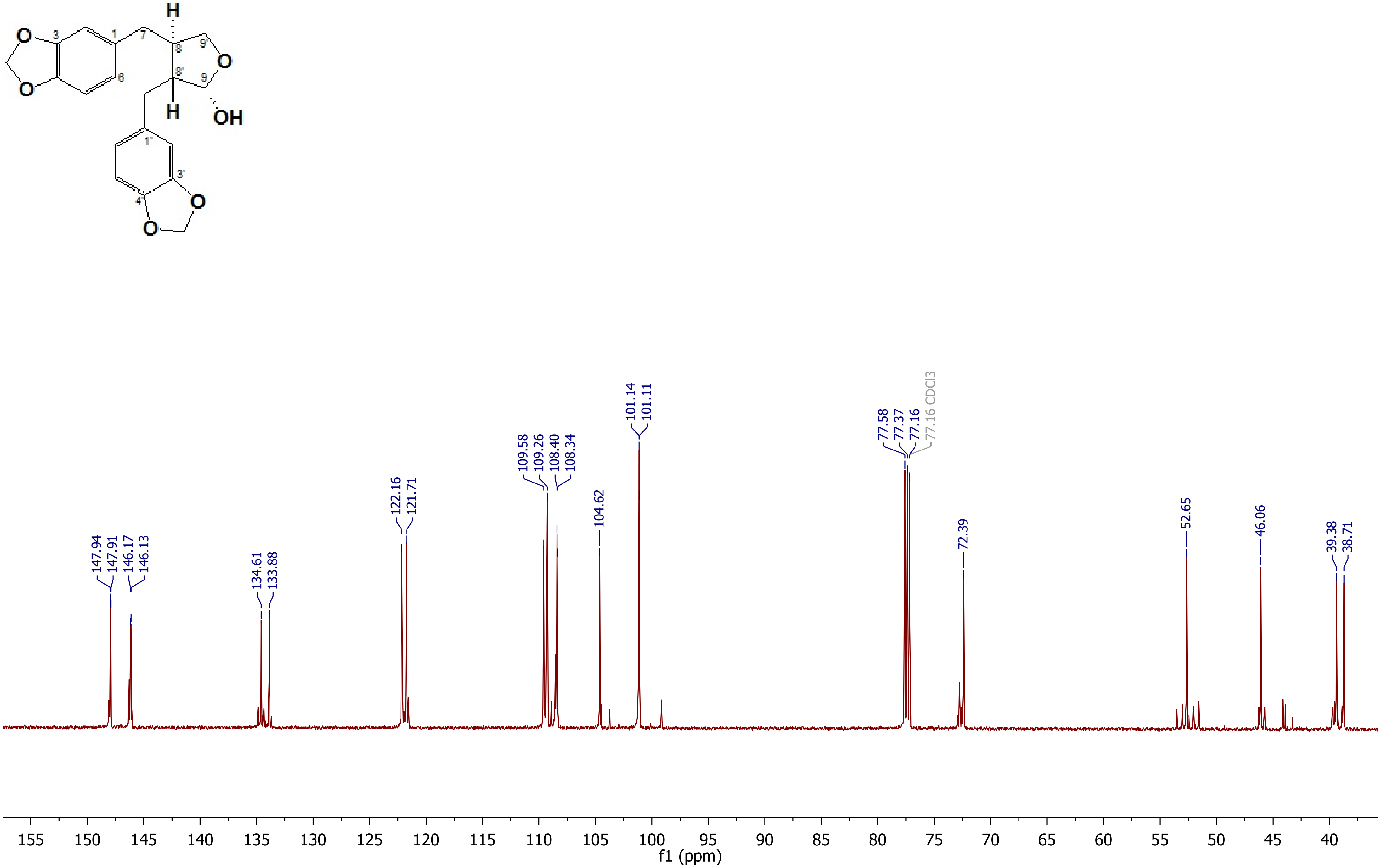

Figure S14. ^{13}C NMR ($CDCl_3$, 150 MHz) Compound **3**.

DEPTQ

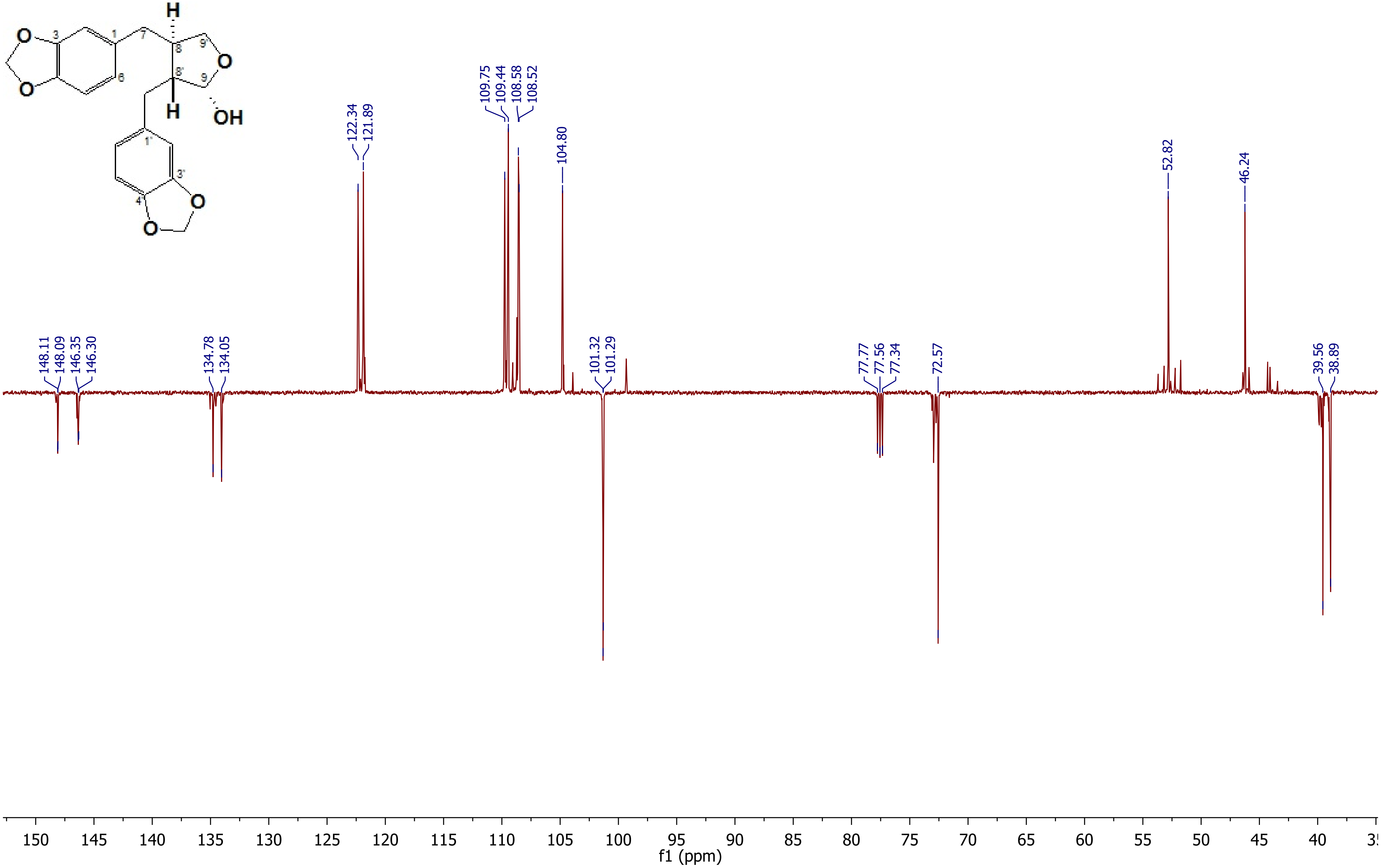

Figure S15. DEPTQ($CDCl_3$, 150 MHz) Compound **3**.

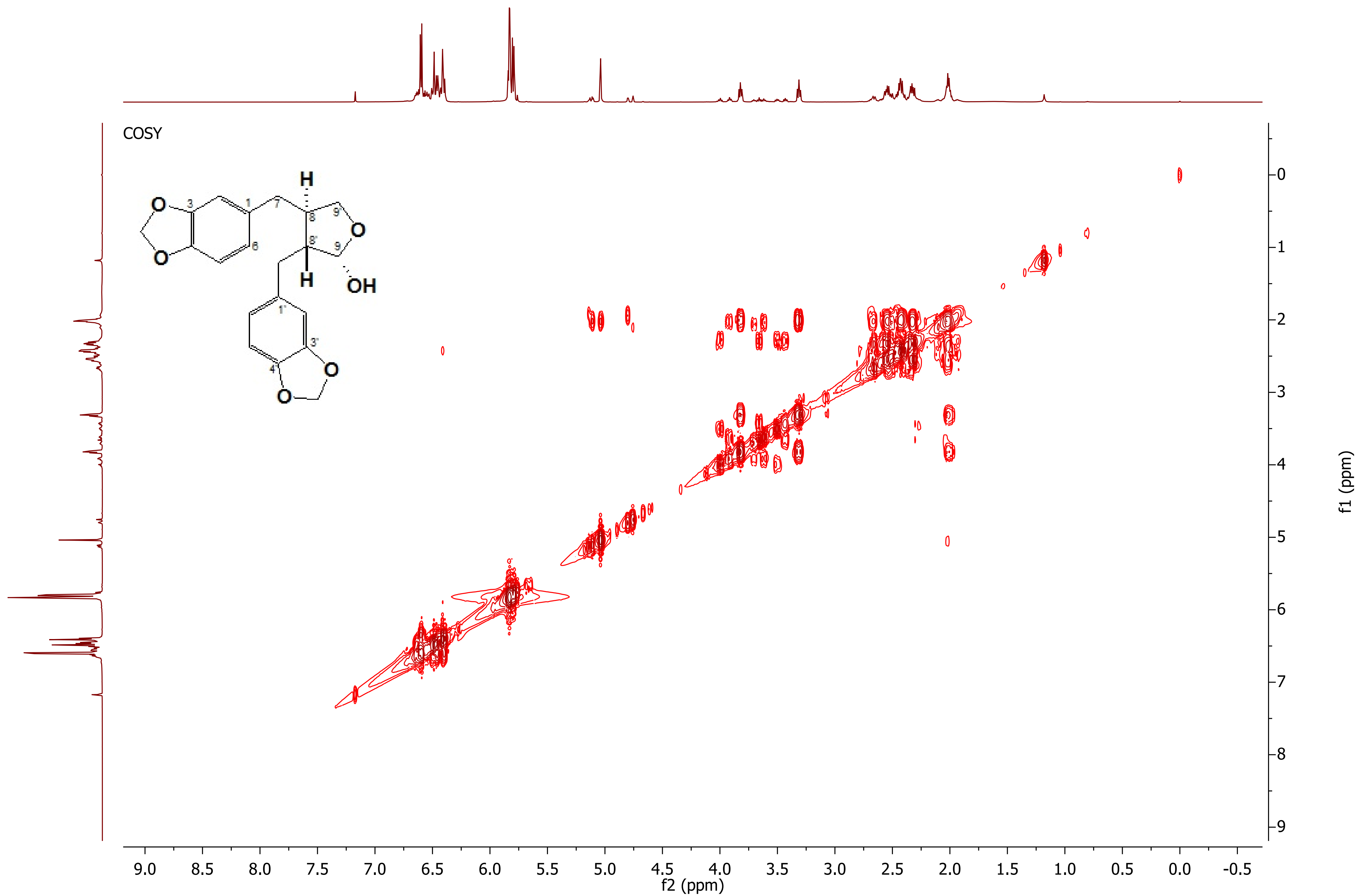

Figure S16. ^{1}H-^{1}H COSY NMR ($CDCl_3$, 600 MHz) Compound **3**.

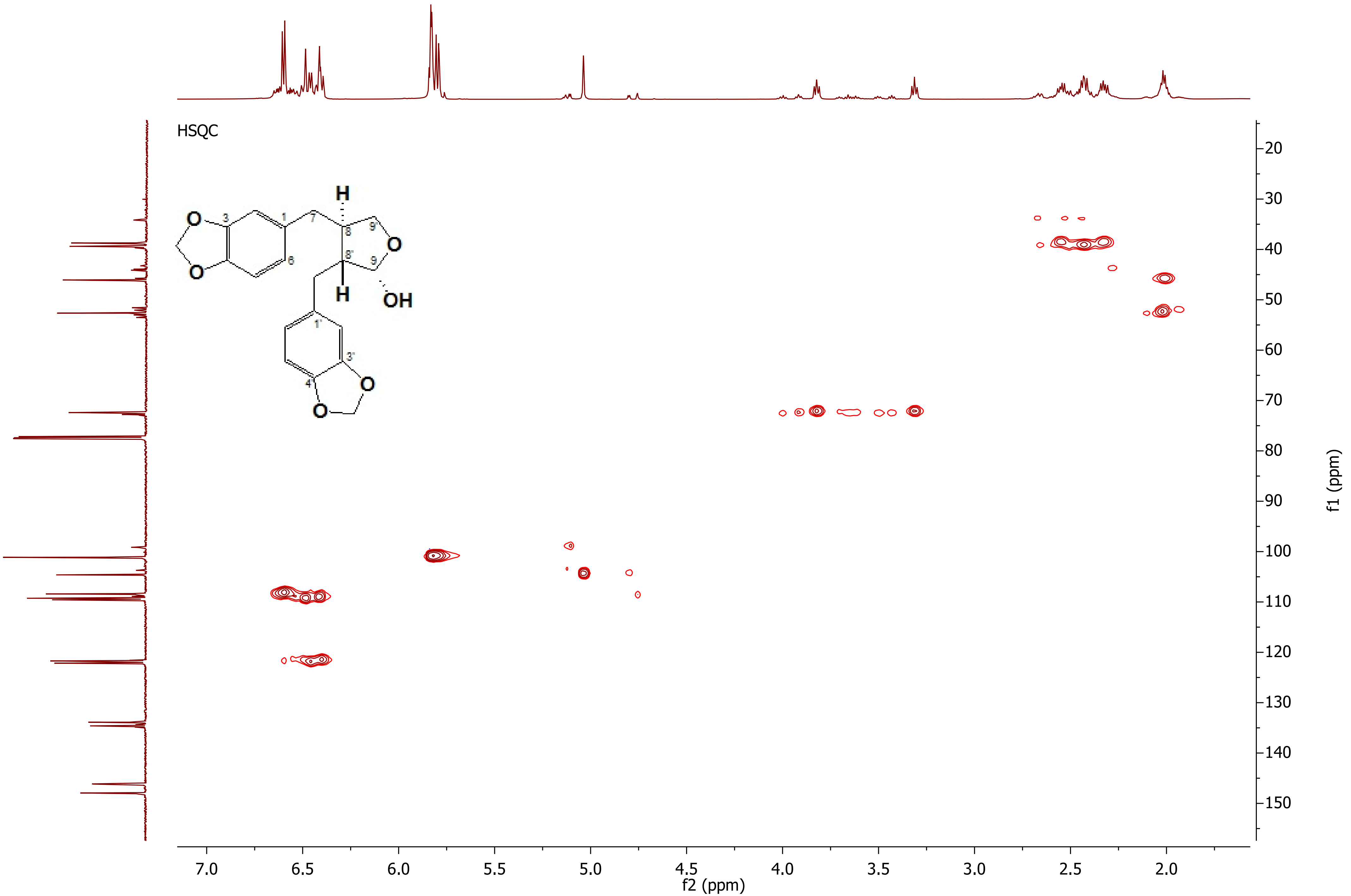

Figure S17. HSQC ($CDCl_3$, 600 MHz) Compound **3**.

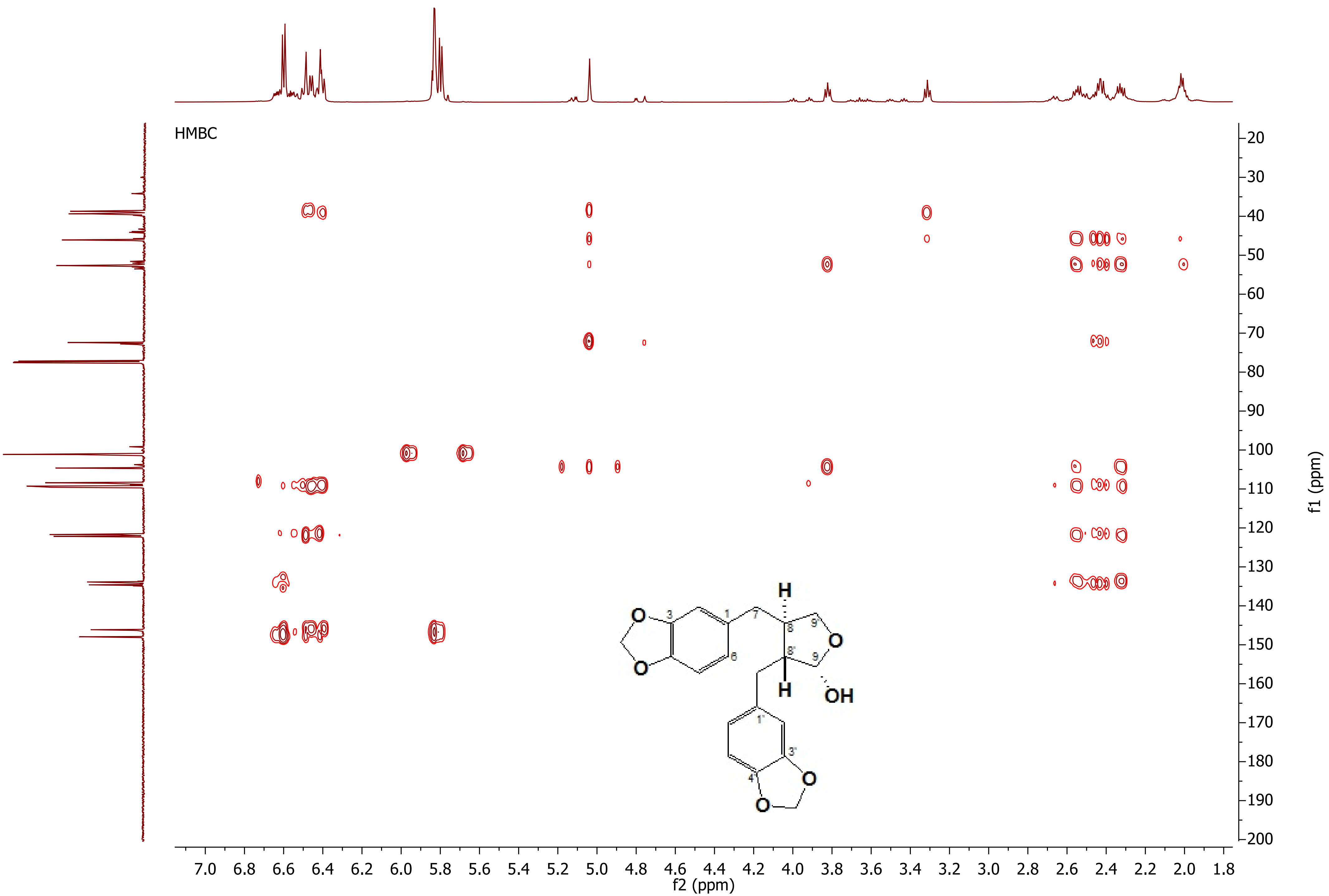

Figure S18. HMBC ($CDCl_3$, 600 MHz) Compound **3**.
